# Criminal Rehabilitation Through Medical Intervention: Moral Liability and the Right to Bodily Integrity

**DOI:** 10.1007/s10892-014-9161-6

**Published:** 2014-04-29

**Authors:** Thomas Douglas

**Affiliations:** Oxford Uehiro Centre for Practical Ethics, Faculty of Philosophy, University of Oxford, Suite 8, Littlegate House, St Ebbes Street, Oxford, OX1 1PT UK

**Keywords:** Bodily integrity, Chemical castration, Consent, Corrections, Criminal rehabilitation, Moral liability, Parole

## Abstract

Criminal offenders are sometimes required, by the institutions of criminal justice, to undergo medical interventions intended to promote rehabilitation. Ethical debate regarding this practice has largely proceeded on the assumption that medical interventions may only permissibly be administered to criminal offenders with their consent. In this article I challenge this assumption by suggesting that committing a crime might render one morally liable to certain forms of medical intervention. I then consider whether it is possible to respond persuasively to this challenge by invoking the right to bodily integrity. I argue that it is not.

Criminal offenders are sometimes required to undergo medical interventions intended to facilitate their rehabilitation.^1^ For example, drug-addicted offenders may in some jurisdictions be required to take medications that replace their drug of addiction and thus remove one potential impediment to rehabilitation.^2^ Similarly, sex offenders are sometimes required to receive injections of testosterone-lowering drugs. These are intended to suppress their sex drive and thus diminish their motivation to sexually offend, or to enhance the effectiveness of psychological rehabilitation programmes.^3^

The use of medical interventions for such rehabilitative purposes has, to date, been limited. However, we might expect it to become more prevalent in the future, since the range of medical interventions capable of facilitating rehabilitation is likely to expand. Behavioural and social neuroscience are currently thriving areas of research and are beginning to uncover neural correlates of dispositions towards aggression, impulsiveness, diminished empathic ability and psychopathy. They are also already suggesting means of influencing these dispositions in ways that might be thought conducive to rehabilitation in some offenders. For example, some widely used antidepressants have recently shown promise in reducing aggression, while the drug divalproex has been found to reduce impulsiveness in adolescents with explosive temper (Bond 2005; Nevels et al. 2010; Donovan 2000; Khanzode et al. 2006).

Given developments such as these, it seems possible that, in the future, we will have available a significant range of medical interventions capable of aiding rehabilitation. It is unsurprising, then, that there is a burgeoning debate on the question whether and when it might be permissible for the state to require that criminal offenders undergo such interventions (Rosati 1994; Bomann-Larsen 2013; Ryberg 2012; Ryberg and Petersen 2013; Vincent 2014; Shaw 2014; Bublitz and Merkel 2014; Douglas et al. 2013; McMillan 2013).

## Preliminaries

The aim of this article is to begin to challenge an assumption that has underpinned much of this debate. Before introducing this assumption, however, I need to define the scope of the discussion by distinguishing some different ways in which medical interventions may be imposed on criminal offenders. Three distinctions will be helpful.

The first concerns the legal context within which requirements to undergo medical interventions are imposed. We can distinguish here between cases in which the requirement to undergo the intervention is imposed with the backing of medical law, for example, mental health or public health legislation, and cases in which it is imposed under the provisions of the criminal law, where this is interpreted broadly so as to include, for example, laws and regulations regarding criminal sentencing and parole.

The second distinction concerns the relevance of the offender's history. In some cases, the imposition of these interventions is triggered solely by forward-looking considerations such as the offender’s future risk to others; it is not linked in any way to past criminal offending, except insofar as this constitutes evidence for future risk. In other cases, however, medical interventions are imposed in part in direct response to the commission of a crime. That is to say, the mere fact that the offender has committed a crime triggers—or is one of a number of considerations that jointly trigger—the imposition of the medical intervention. This article, focuses on medical interventions that are imposed on criminal offenders, for the purpose of facilitating rehabilitation, (1) under the provisions of the criminal law, and (2) in direct response to the commission of a particular crime. I will refer to medical interventions used in these ways as *medical correctives*.^4^

A third distinction has played an important role in ethical debate regarding the permissibility of imposing medical correctives. It concerns the avoidability of the requirement to undergo the corrective. When medical correctives have been used to date, they have frequently been imposed as a condition of parole or early release. Thus, the offender can escape the requirement by electing to remain in prison. He is effectively presented with a choice between receiving the medical corrective, and enduring further incarceration. In other cases, however, the requirement to undergo the medical intervention is unavoidable. The medical corrective may, for example, be included as a compulsory element of a criminal sentence.

Most scholarly debate has focussed on the imposition of medical correctives as a condition of parole or early release, which has been seen as the less controversial approach. However, even this practice has been heavily criticised, most often on the ground that, when the only alternative is to remain incarcerated, an offender’s choice to receive a medical corrective is coerced, and this renders his consent to it invalid. For instance, Kari Vanderzyl argues that the practice of offering sex offenders the choice between undergoing testosterone-lowering interventions—either chemical or surgical castration—as an alternative to incarceration is ‘inherently coercive’:

the doctrine of informed consent requires a knowledgeable and voluntary decision to undergo treatment, yet offering a convicted offender castration as an alternative to a lengthy prison sentence constitutes an inherently coercive practice rendering truly voluntary consent impossible. Thus, castration should be rejected as a condition of probation (Vanderzyl 1994–1995: 140).

Similarly, in relation to the chemical or surgical castration of convicted rapists, William Green maintains that

Voluntary consent depends upon a person’s ability to make a choice freely··· The convicted rapist is faced with two options—a lengthy prison sentence or even death on the one hand and Depo-Provera [a form of chemical castration] or surgical castration on the other—and cannot be said to have the capacity to act freely in making a choice. Freedom of choice is impossible because the convict’s loss of liberty constitutes a deprivation of such a magnitude that he cannot choose freely and voluntarily, but he is forced to give consent to an alternative he would not otherwise have chosen. In such circumstances men are willing to “barter their bodies.”… As a consequence, the convicted rapist cannot give voluntary consent to an offer of probation which contains a surgical castration or Depo-Provera condition (Green 1986, 16–17).

The dominant response to these claims has been to argue that, though offenders offered a choice between undergoing medical correctives and further incarceration clearly face pressure to consent to castration, that pressure does not render their consent invalid, for example, because it does not amount to coercion, or does not undermine autonomy (e.g., Rosati 1994; Ryberg 2012; Ryberg and Petersen 2013; Wertheimer and Miller 2013). Proponents of this response have typically accepted, at least implicitly, an assumption made by those to whom they are responding, namely:

*The Consent Requirement*: Medical correctives can only permissibly be provided with the valid consent of the offender who will undergo the intervention.

In this article, I wish to challenge this assumption and thus lay open an alternative line of response. I do not attempt to conclusively refute the Consent Requirement. But I do seek to show that it is more difficult to defend than might initially be thought, and than has, I believe, widely been assumed by both opponents and proponents of the use of medical correctives. I attempt to do this by first outlining a problem faced by the proponent of the Consent Requirement, and then arguing that what I take to be the most obvious response to this problem—an appeal to the right to bodily integrity—runs into difficulties of its own.

Throughout I assume, to keep things manageable, that all medical correctives consist in the injection of a drug—that is, a biologically active, non-food substance. I thus limit the scope of the Consent Requirement so that it applies only to medical correctives of this kind. I suspect that many defenders of the Consent Requirement would be prepared to extend it to cover less invasive medical interventions, such as the oral administration of drugs, but I do not assume that they would do so here. Similarly, it should not be assumed that, in challenging the Consent Requirement, I am defending the view that more invasive interventions—such as major surgical procedures—could permissibly be imposed without consent.

## The Problem of Moral Liability

The scope of the Consent Requirement is, then, limited such that it covers only medical interventions that consist in the injection of a drug. As I formulated it above, it is also limited in another way. It covers only medical *correctives*: medical interventions that are provided as part of the criminal justice system’s response to the commission of crime, and for the purposes of facilitating rehabilitation. However, the Consent Requirement, thus understood, could be regarded as an extension of a comparable requirement regarding the use of medical interventions for conventional therapeutic purposes within ordinary clinical contexts. It is now generally taken as beyond dispute that such therapeutic medical interventions should not, except in certain special circumstances, be provided to a competent adult patient without consent, and it is natural to think that a parallel claim would hold with respect to medical correctives.

There is, however, a potential difficulty with extending this requirement for consent from orthodox therapeutic medical interventions to medical correctives: it is widely thought that the state may permissibly do things to criminal offenders without their consent that it could not permissibly do to others without (and in some cases even with) consent. Thus, for example, it would ordinarily be grossly wrong to incarcerate someone without consent, but in the context of criminal justice, nonconsensual incarceration is widely thought to be permissible. Nonconsensual incarceration may be hard to justify given prevailing prison conditions, which often involve exposing incarcerated individuals to overcrowded conditions, a high risk of rape and assault, and serious health threats (Stern 2001; Human Rights Watch 2001; Wolff et al. 2007; Wolff and Shi 2009). But we can imagine other, less harsh arrangements that would nevertheless deserve the name "incarceration". Suppose that offenders could be held in institutions that placed serious and constant constraints on free movement and association, but otherwise exposed offenders to no greater risks to their health and security than average members of the unincarcerated citizenry, and took all reasonable steps to safeguard opportunities for political participation, legal representation and education. It would be widely (though not universally) accepted that the state could permissibly impose conditions of this sort—henceforth, ‘minimal incarceration’—on at least some criminal offenders.

Plausibly, in committing certain crimes, an offender becomes morally liable to the imposition of minimal incarceration, and for a substantial period. (In what follows, I will simply assume that this is so.) This raises the question whether they might also become liable to the imposition of some varieties of medical intervention. Indeed, given the rather drastic effect of criminal offending on the range of interventions to which one is liable—these interventions plausibly also include psychological rehabilitation programmes, fines, community service, probation regimes, and the freezing of financial assets—it might seem that a proponent of the Consent Requirement owes us an explanation as to why medical interventions are not among the interventions to which we become liable. We might ask: what sets medical intervention apart from minimal incarceration and the other kinds of intervention to which criminal offenders become liable? Why is it that, following offending, consent is required for the imposition of medical correctives, but not for these more traditional kinds of criminal remedy?

In what follows, I will focus on the comparison between medical correctives, on the one hand, and minimal incarceration, on the other. I will consider whether and how one could justify the Consent Requirement, which rules out the nonconsensual imposition of medical correctives, while rejecting any comparable constraint on the nonconsensual imposition of minimal incarceration.

There are several differences between medical correctives and minimal incarceration to which one might plausibly appeal here. One possibility would be to point to differences in the purposes for which incarceration and medical correctives would be imposed. Medical correctives are, we are assuming, employed in order to aid the offender’s rehabilitation. By contrast, incarceration, it might be argued, is intended to mete out deserved suffering, to communicate social disapproval, or to deter third parties from offending. Thus, one might argue that consent is required for medical correctives, but not for minimal incarceration, on the grounds that the goals of incarceration are different to, and perhaps morally more urgent than, the goals served by medical correctives. Perhaps it is permissible to nonconsensually treat offenders in intrusive ways in order to realise retributive, communicative or deterrent goals, but not in order to realise rehabilitative ones.

However, rehabilitation—the goal for which medical correctives are imposed—has commonly also been regarded as a goal, and in some cases, the only goal, of incarceration.^5^ Moreover, two other goals sometimes attributed to incarceration—incapacitation and deterrence—are commonly thought to serve the same higher objective as rehabilitation: namely, the prevention of crime or, more generally, the maintenance of security. Thus, it seems worth considering what would follow for the Consent Requirement *if* the goal of rehabilitation—or whatever higher goal rehabilitation serves—were sufficiently important that it could justify the nonconsensual imposition of minimal incarceration. In such a case, one might wonder how the same goal could fail to justify the nonconsensual imposition of at least some medical correctives.

In what follows I will thus consider whether it is possible to defend the Consent Requirement even on the assumption that the ultimate goal for which medical correctives are imposed is sufficiently important that it could justify nonconsensual minimal incarceration. I examine only what I take to be the most obvious defence of this kind—and the defence which I suspect the authors quoted above would invoke if pressed. This defence appeals to a right to bodily integrity.

## The Right to Bodily Integrity

If we possess any moral rights at all, it is plausible that a right to bodily integrity is among them. This I take to be a right that protects against intentional interference with one’s body, or certain kinds of such interference. An important feature of this right is that it protects against the relevant kinds of bodily interference regardless of what consequences that interference might contingently have, and regardless of what motives might contingently have motivated it. It is a right against bodily interference as such. Suppose, for example, that Smith pins down Jones and severs his hand despite Jones’ strident and reasonable protests. Whether Smith does this in order to save Jones’ life (say, because Jones’ hand is gangrenous) or in order to achieve vengeance as part of a family feud is irrelevant to the question whether Smith violates Jones’ right to bodily integrity, though it may be relevant to the question whether he justifiably does so. Similarly, whether severing Jones’ hand will in fact save his life, and whether it can be *expected* to do so make no difference to whether Smith violates Jones’ right to bodily integrity. The right to bodily integrity is insensitive to such differences.

It is plausible that the right to bodily integrity typically rules out the nonconsensual imposition of medical interventions, at least, medical interventions which are, like the injection of drugs, physically invasive. Certainly, appealing to a right to bodily integrity is a standard way of defending the requirement that medical interventions, when used therapeutically within the context of clinical medicine, should be used only with the consent of the patient.^6^ It might seem, furthermore, that the right to bodily integrity will rule out the nonconsensual imposition not only of orthodox therapeutic medical interventions, but also of medical correctives. After all, imposing a medical intervention as a medical corrective constitutes bodily interference of the same kind as does imposing a medical intervention for therapeutic purposes, and though the motives and perhaps consequences associated with this interference will differ between clinical and forensic contexts, the right to bodily integrity is, as we have just seen, insensitive to such differences. On the other hand, it seems doubtful whether the right to bodily integrity could be invoked against the nonconsensual incarceration of criminal offenders, for incarceration does not obviously involve bodily interference: it involves the placement of barriers external to the body. Thus, an appeal to a right to bodily integrity seems a *prima facie* promising way of defending the Consent Requirement without committing oneself to the impermissibility of nonconsensual incarceration.

It is, however, possible to challenge this defence. An initial difficulty is that the sorts of considerations which support the existence of a right to bodily integrity might also be thought to support the existence of rights to free movement and free association, and these rights might seem to create trouble for the view that nonconsensual incarceration is permissible. For example, one reason to suppose that there is a right to bodily integrity is that nonconsensual interference with the bodies of innocent persons seems, to many, to be seriously wrong in most cases, even where it seems that it could be justified on utilitarian grounds. A right to bodily integrity could account for this wrongness, and this, arguably, lends credence to the view that there is such a right. But similar considerations could be invoked in support of a right to free movement and association, for nonconsensually constraining an innocent person’s freedom of movement and association—for example, through incarceration—also seems to many to be seriously wrong in most cases, even where it might seem justifiable on utilitarian grounds. For example, knowingly imprisoning an innocent person is abhorrent to most, even where it would successfully deter many grave crimes.

A second reason to suppose that there might be a right to bodily integrity is that the existence of such a right might seem to be entailed by the existence of a more basic right to self-determination. It might seem that, given the crucial dependence of the self on the body, interfering with the body involves interfering with self-determination. Again, however, similar considerations would support the existence of rights to free movement and association, since severely constricting a person’s freedom of movement and association just as plausibly involves interfering with that person’s self-determination.

Parallels such as these cast doubt on the possibility that one could consistently posit a right to bodily integrity without also positing a right to free movement and association. Moreover, even if one could consistently do this, it seems unlikely that many would want to adopt this combination of views: most rights theorists who endorse a right to bodily integrity would also endorse rights to free movement and association, and the existence of these latter rights appears, at least at first sight, to be in tension with the view that incarceration can be permissible. Thus, the appeal to bodily integrity, as specified thus far, does not clearly allow one to deny the permissibility of nonconsensual medical correctives while endorsing nonconsensual minimal incarceration.

At this point it might be argued that the existence of rights to free movement and association can, in fact, be reconciled with the permissibility of incarceration. Indeed, I have already alluded to one way in which such a reconciliation might be achieved. I noted earlier that criminal offending can make one liable to certain forms of treatment that would otherwise be impermissible, including incarceration. *One way* in which criminal offending may do this is by causing one’s rights to free movement and association lose some of their normal protective force following criminal offending.^7^

There are various ways of accounting for this loss of protective force. One explanation would hold that, in offending, one simply waives one’s rights to free movement and association. Another would hold that one activates an exception clause already built into those rights. Yet another would maintain that, in offending, one confers on others a right to restrict one’s movement and association, and this right must then be balanced against one’s persisting rights against such restrictions. But however we account for the change, it seems that there *is* a change: if people normally possess rights to free movement and association, yet the incarceration of criminal offenders can be justified, then criminal offending must cause the rights to free movement and association to lose their normal protective force.

At this point, however, a further difficulty arises: if criminal offending causes rights to free movement and association to lose some of their protective force, we might well wonder whether it has a similar effect on rights to bodily integrity. Perhaps offending causes one’s right to bodily integrity to lose some of its protective force, making it the case that certain nonconsensual medical interventions are no longer impermissible. Thus, even if we grant that the right to bodily integrity normally rules out nonconsensual medical intervention, it might fail to rule out the nonconsensual imposition of medical interventions *as medical correctives.* The appeal to bodily integrity thus faces a more specific version of the problem of moral liability discussed in Sect. 2.

However, once the right to bodily integrity is on the table, it becomes easier to see how one might respond to this problem. One might invoke the *prima facie* plausible view that the right to bodily integrity is more robust than rights to freedom of movement and association.^8^ The rough idea here is that it takes a greater deviation from normal circumstances for the right to bodily integrity to lose its protective force than for the rights to freedom of movement and association to lose their protective force; it takes more to render oneself liable to impositions on bodily integrity than to render oneself liable to impositions on free movement and association.^9^ If this is correct, then, even though committing a crime makes one liable to the restrictions on free movement entailed by incarceration, it may not make one liable to impositions on bodily integrity of the sort entailed by compulsory medical remedies. The right to bodily integrity may remain in place, and retain its normal protective force.

In what follows, I consider whether this response succeeds. I consider, that is, whether the right to bodily integrity is indeed more robust, in the relevant sense, than rights to free movement and association. However, first, I need to sharpen the response by specifying the relevant rights, and the relevant dimensions of robustness, more fully.

## The Robustness Claim

The right to bodily integrity is most naturally thought of as a general right that protects against a wide range of different kinds of treatment. For example, it may protect its bearer against many different kinds of nonconsensual bodily interference ranging from relatively innocent forms of physical contact to major surgical procedures and extreme forms of physical violence. However, it is possible to think of this general right as being composed of more specific rights against specific kinds of bodily interference. Moreover, it is plausible to think that these more specific rights might differ in their robustness. We might expect that we possess a more robust right to bodily integrity in respect of extreme physical violence than in respect of, say, non-sexual touching. This is why it takes more to become liable to extreme physical violence than to become liable to non-sexual touching.

Similar thoughts apply to rights to free movement and association. These are most naturally thought of as general rights that protect against a wide variety of restrictions on movement and association. However, we can think of these general rights as being composed of more specific rights against particular kinds of restriction, and we might think that these more specific rights will differ from one another in their robustness.

The proponent of the line of argument that I wish to consider here is, as I will understand her, interested in comparing the robustness of one *specific* right to bodily integrity with that of one *specific* right to free movement and association. On the one hand, she is interested in the right to bodily integrity that protects against the injection of a drug (henceforth simply ‘injection’).^10^ On the other hand, she is interested in the specific rights to free movement and association that protect against the kinds of constraints on movement and association involved in minimal incarceration. Our imagined interlocutor maintains that the specific rights to bodily integrity that protect against injection are more robust than the specific rights to free movement and association that protect against the kinds of restrictions entailed by minimal incarceration.

She maintains, moreover, that these rights are more robust on one dimension in particular: they are more robust *in the face of criminal offending*. There are various kinds of deviation from normal circumstances that may cause a right to lose its protective force, and these correspond to different dimensions of robustness. Our interlocutor is interested in one kind of deviation, namely, the commission of a crime by the rightholder. She asks ‘how serious must an individual’s criminal offending be before her rights to bodily integrity, free movement and free association lose their protective force?’ And she answers that it takes more serious criminal offending for the right to bodily integrity to lose its protective force than for the other two rights to lose theirs. Indeed, if she wishes to argue that the right to bodily integrity *always* rules out the nonconsensual imposition of medical correctives, she will need to maintain that *however serious* one’s criminal offending, one’s right to bodily integrity retains its protective force. But I will not attribute this view to her here. I attribute to her only

*The Robustness Claim*: It takes *more serious* criminal offending for the rights to bodily integrity that protect against injection to lose their protective force than for the rights to free movement and association that protect against minimal incarceration to lose theirs.

Except where otherwise specified, I henceforth take ‘robustness’ to mean ‘robustness in the face of criminal offending’ and I use the phrases ‘rights to bodily integrity’ and ‘rights to free movement and association’ to refer to the specific rights mentioned in the Robustness Claim. This allows me to paraphrase that claim simply as:

Rights to bodily integrity are more robust than rights to free movement and association.

The Robustness Claim gains some initial credibility from the observation that committing a crime does not render one liable to *all* impositions of the sort that would otherwise plausibly violate one’s rights. For example, it does not, I would say, make one liable to killing, torture or public humiliation. We plausibly possess some rights that are more robust than rights to free movement and association such that they would retain their protective force even if we committed serious crimes. Thus, the proponent of the Robustness Claim need not argue that rights to bodily integrity are exceptional in any way. She need only argue that they are, in respect of their robustness, more like rights against torture, killing and public humiliation than they are like rights to free movement and association.

Nevertheless, I think it is doubtful whether an appeal to the robustness of the right to bodily integrity can succeed in ruling out the possibility that criminal offending makes one liable to the imposition of medical interventions. Though there are plausibly some rights that are more robust in the face of criminal offending than rights to freedom of movement and association, it is, I think, difficult to see why we should place the right to bodily integrity among them. To buttress this point, I now turn to consider two strategies via which one might attempt to establish the Robustness Claim. I argue that each faces serious difficulties.

## Appeals to Intuition

An initial strategy would be to appeal to case-based intuitions. The simplest version of this strategy would appeal to intuitions about cases involving criminal offending. If we could identify cases in which, intuitively, it would be permissible to subject an offender to minimal incarceration, but not to an injection, we would arguably have some intuitive support for the view that rights to bodily integrity are more robust than rights to free movement and association. This approach faces two problems, however. First, it lacks dialectic force against the advocate of medical correctives. Such an advocate is, I take it, unlikely to share the relevant intuitions. Second, it appeals to intuitions whose evidential status is uncertain. Insofar as we find the imposition of minimal incarceration more acceptable than the imposition of an injection in such cases, this may simply reflect our much greater familiarity with incarceration than medical interventions within the realm of criminal justice. But if our intuitions in this area are driven by differences in familiarity, then they will have no evidential value in respect of the moral questions of interest to us here.

There are, however, other cases to which one might appeal in order to provide less direct intuitive support for the Robustness Claim. I noted above that there might be other factors, besides criminal offending, that can cause rights to bodily integrity, free movement and free association to lose their protective force. If one could show that, intuitively, rights to bodily integrity are more robust in the face of these other factors than rights to free movement and association, this might provide indirect support for the view that they will also be more robust in the face of criminal offending, for it might support the view that they are *generally* more robust. Thus, for example, it is plausible that both rights to bodily integrity and rights to free movement and association can lose their protective force in the face of potential catastrophes: it may be permissible to interfere with someone’s body, or to constrain his movement and association, if this is necessary to avert a catastrophe. But suppose it could be shown that, intuitively, it takes a much more serious potential catastrophe to justify bodily interference than to justify restrictions on movement and association. We would then have some reason to believe that rights to bodily integrity are more robust in the face of potential catastrophes than rights to free movement and association, and insofar as this supports the view that the former rights are quite generally more robust than the latter, this will indirectly support the view that they are more robust in the face of criminal offending as well.

The difficulty with this more indirect approach, however, is that, when we look to cases in which criminal offending plays no role, it is difficult to find clear intuitive support for the view that rights to bodily integrity *are* more robust than rights to free movement and association. There are, admittedly, some cases regarding which some might have intuitions supportive of the Robustness Claim. Consider the following pair of cases:

*Jill* is infected with a novel strain of the Ebola virus, which could, if it spread, infect and kill many people. The only way to stop it spreading is to keep her in quarantine for three months, and, since Jill does not agree to this, the quarantine would have to be imposed against her will.

*Jane* is infected with a novel strain of the Ebola virus which could, if it spreads, kill many people. The only way to stop the virus spreading is to inject Jane with a drug. This drug will not cure Jane’s infection, but it will prevent the virus from infecting others. Jane does not agree to receive the injection, so it will need to be imposed against her will.

Here we are considering imposing constraints on Jill’s movement that are similar to those involved in minimal incarceration. We are also considering interfering with Jane’s body through the imposition of an injection.

It might perhaps seem plausible that, if the threat to the public health is great, both of these interventions could be permissible, especially if the negative side-effects thereby inflicted on Jill and Jane would be modest. However, some might intuitively judge that our threshold for imposing the safe injection should be higher than our threshold for imposing the period of quarantine, holding fixed all unspecified factors (such as the severity of the side-effects of the two interventions). They might judge, that is, that a greater public health threat would be required to justify the injection than to justify the period of quarantine. This might seem to support the view that rights to bodily integrity are more robust than rights to free movement and association in the face of pandemic threats.

My own intuitions about these cases run in the opposite direction. It seems to me that, other things being equal, the threshold for imposing the period of quarantine should be at least as high as the threshold for imposing the safe injection. However, I will not attempt to make anything of this intuitive response here. Instead, I will settle for the (I hope rather uncontroversial) claim that it is not intuitively *clear* that the threshold for intervention should be higher in the case of Jane than in the case of Jill. If this is correct, then any intuitive support for the Robustness Claim provided by this case will be weak.

Similar thoughts apply to cases, outside of the context of criminal justice, where competent individuals pose a threat to themselves or to others as a result of a severe mental disorder. Many jurisdictions allow such individuals to be subject either to forced commitment to a psychiatric institution (a restraint that is comparable to incarceration with respect to freedom of movement and association) or to forced treatment (which frequently involves bodily interference of the same sort as, we are supposing, is involved in the imposition of a medical corrective). In these cases, as in cases of pandemic control, the fact that an individual poses a threat to others appears to cause her rights to bodily integrity, free movement and free association, to lose their normal protective force. But again, it is, I think, not intuitively clear that a greater threat to others should be required in order to impose forced psychiatric treatment (without containment) than to impose containment without treatment. Indeed, interestingly, the laws in many jurisdictions adopt the same threshold for treatment as for containment.

It may, of course, be possible to find cases in which rights to bodily integrity do seem, intuitively, to be clearly more robust, on some dimension, than rights to free movement and association; that I find it difficult to imagine such cases does not show that they do not exist. However, the difficulty identifying such cases at least casts doubt on the suggestion that the Robustness Claim can be defended by an appeal to intuitions about such cases.

## Theoretical Considerations

At this point, the proponent of the Robustness Claim might turn to an alternative strategy. I noted above that there seem to be *some* rights, such as rights against killing, torture and public humiliation, that clearly are more robust in the face of criminal offending than rights to free movement and association. Many would hold that, however serious or frequent one’s criminal offending, *these* rights never lose their protective force. Perhaps one could argue that the considerations which explain why these rights are highly robust, and more robust than rights to free movement and association, apply also to rights to bodily integrity.

### Harm

One consideration that many would appeal to in seeking to explain the great robustness of these rights is the fact that they protect against forms of treatment that typically cause very serious harm, more serious harm than constraints on free movement and association of the sort involved in minimal incarceration. Certainly, it is plausible that killing is typically more harmful than restricting someone’s freedom of movement and association. However, it is difficult to see how this consideration could be invoked to show that rights to bodily integrity are more robust than rights to free movement and association. The restrictions on movement and association entailed by incarceration—even minimal incarceration—would reliably cause (and may themselves constitute) significant harms. They would frequently damage existing personal relationships while making it difficult to form new ones, they would seriously restrict sexual freedoms, they would make it impossible to pursue most careers, and they would more generally prevent the realisation of many life-plans. Moreover, even in cases where minimal incarceration did not, by chance, seriously frustrate life plans or relationships, we might expect it to cause significant distress. After all, imposing more extreme forms of confinement and social isolation is an established cause of severe distress and has frequently been used as a punishment or torture technique for precisely this reason.^11^

All things considered, then, constraints on free movement and association of the sort involved in minimal incarceration are likely to be severely harmful in most cases. It is, I think, difficult to see why we should expect that the imposition of an injection would normally inflict *more* harm. Many drugs have only relatively minor negative side-effects. It might be argued that much of the harm typically caused by nonconsensual bodily interference stems not from the biologically mediated effects of the interference, but consists rather in the way in which the act of interference is experienced by the victim. Most people attach great symbolic value to their bodies and experience nonconsensual interference with their bodies as highly intrusive and often disrespectful.^12^ As a result bodily interference can inflict a great deal of harm even when its biologically mediated effects are relatively minor. This is perhaps clearest in cases of rape. Nir Eyal asks us to imagine a case in which a rapist carries out a rape in such a way that it causes no physical pain and there is no risk of pregnancy (or, let us add, infectious disease) for the victim. As Eyal notes, we would of course condemn such a rape. Moreover, we are likely to do so at least partly on the ground that, notwithstanding the lack of biologically mediated adverse effects, it is likely inflict great harm. It is likely to be experienced as intrusive, degrading and humiliating, and for these reasons among others, to inflict great psychological pain.^13^

As cases of rape demonstrate, bodily interference can, even if medically safe, be seriously harmful because of the way the interference is experienced (as I will say, it can cause substantial *experiential* harm). However, there is scope to question whether bodily interference *of the sort we are interested in here* would typically be more harmful than minimal incarceration for this reason. After all, not all forms of nonconsensual bodily interference are experienced in ways that cause great pain. For example, absent a sexual context or uneven power relationship, forms of touching such as a hand on the shoulder are typically not experienced as highly distressing, at least, not in many cultures. Rape is arguably unusual among forms of bodily interference in the degree of experiential harm it causes.

The relevant question here is whether the nonconsensual injection of a drug is likely to be experienced in such a way as to cause substantial psychological pain—substantial enough to make it more harmful, all things considered, than restrictions on free movement and association of the sort involved in minimal incarceration. I am not aware of any social scientific evidence that would allow us to answer this question with confidence. However, there is, I think, some reason to doubt that nonconsensual injection is typically experienced so negatively. Very large numbers of people are already subjected to injections that are either compulsory, or at the very least costly to avoid. I am thinking here of children, young adolescents and healthcare workers who are required, or strongly encouraged, by their parents or employers to participate in routine vaccination programmes. If such widespread practices were typically experienced in seriously negative ways—negative enough that they are more harmful, all things considered, than minimal incarceration—it would be rather surprising that this has not been noticed and has not become the subject of extensive research and public debate.^14^ (Contrast, for example, the voluminous literature on the way in which coerced or unwanted sexual encounters are experienced.) This would be particularly surprising given that the *medical* consequences of vaccination programmes have been widely studied and debated, so it is not simply the case that vaccination programmes have been introduced without thought being given to their possible harmful effects.

### Threats to Agency

A second consideration that may justify the great robustness of rights such as those against killing, torture and public humiliation has a more Kantian flavour. It might be argued these rights are highly robust because they protect against forms of treatment that constitute, in some sense, an attack on the victim’s agency. Killing, torture and public humiliation are all forms of treatment that, many would argue, threaten agency in a way that constraining someone’s free movement and association does not.

We can distinguish two different ways in which they may constitute a threat to agency. First, when *A* kills, tortures or publicly humiliates *B*, this may express a denial of, or at least disregard for, *B*’s agency. It may express the proposition that *B* is not an agent, or at least, that *A* does not care whether *B* is an agent or not. Let us call this type of threat a *communicative* threat to agency. Second, when *A* kills, tortures or publicly humiliates *B*, this may constitute an attack on *B*’s agency in the more straightforward sense that it will, or can be expected to, interfere with or diminish *B*’s agency, or sense of agency. Call this a *causal* threat to agency.^15^

It seems somewhat plausible that rights against killing, torture and public humiliation are so robust because killing, torture and public humiliation all involve, or at least typically involve, serious communicative or causal threats to agency. And it might be thought that rights to bodily integrity are highly robust—and more robust than rights to free movement and association—for the same reasons.

For this suggestion to be plausible, however, there will need to be some reason to think that interfering with someone’s body by injecting a drug (typically) constitutes a more grave threat to agency than does constraining his freedom of movement and association in the way entailed by minimal incarceration. Given the distinction between communicative and causal threats to agency introduced above, we can distinguish two different grounds for regarding bodily interference to be a more serious threat to agency than restriction of movement and association. It could be more serious in that it involves a more severe reduction in the victim’s agency or sense of agency. Or it could be more serious in that it expresses a more thoroughgoing denial of or disregard for the victim’s agency.

It is difficult to see why it need be a more serious threat to agency in either of these respects. Arguably, nonconsensual interference with another’s body invariably expresses *some degree* of disregard for the other’s agency, for it involves ignoring the preferences of that individual, and we might take those preferences to express his agency. But the same can be said for nonconsensual restrictions on free movement and association; these also involve ignoring the preferences of the person whose freedom of movement and association is restricted.

Bodily interference *may* also express a more thoroughgoing disregard for the agency of another. If one injects someone with a drug because one believes he is no different from a dangerous animal and should be treated as such, one clearly expresses a serious disregard for his agency. But this is because one’s action is motivated by a set of beliefs and desires which presuppose that the other is not an agent, or ought to be treated as though he is not an agent. Interference with another’s free movement could also be motivated by such desires and beliefs; after all, restriction of movement is also the kind of intervention to which we might subject a dangerous animal. And were we to restrict movement for those reasons, it would, I think, constitute a comparably serious communicative threat to agency. It is, moreover, difficult to see any reason why interference with bodily integrity would be any more likely to be driven by such motives than restrictions on free movement, so it would be difficult even to argue that bodily interference *typically* involves a more serious communicative threat than restrictions on free movement and association.

Similar thoughts apply to causal threats to agency. Bodily interference *could* interfere with agency, for example, by inducing depression or extreme alienation from one’s self. But so too could constraints on free movement and association, and I see no reason to suppose that bodily interference would invariably or more probably produce such effects than restrictions on free movement and association. Agency resides in the mind, and the mind is, of course, dependent on and influenced by the body in various ways. Thus, there is certainly a risk that anything that interferes with our bodies will also interfere with our agency. But constraints on free movement and association can also interfere with our agency. They do so most obviously by removing various options (possible actions) that we might otherwise have chosen to take. An incarcerated individual may still have full agency, where that is understood as a mental capacity, but he cannot fully exercise that capacity because so many possible actions are closed off.

It might be argued that merely removing external options is less threatening to agency than interfering with the mind—and thus with the very capacity for agency. Interference with the mind might be thought a particularly severe threat to agency because of the way in which it interferes with agency at its roots. But here it should be noted that constraints on free movement can also interfere with the mind, and thus, potentially, with the very capacity for agency. After all, the mind is in various respects dependent on, and influenced by, our immediate natural and social environment, which in turn is affected by restrictions on free movement and association. (An incarcerated individual occupies a very different social and natural environment than a free person.) According to the extended mind thesis and some of its close relatives, some parts of our environment are in fact part of our minds, or at least, are on a par with brain states in serving as part of the physical basis for our minds.^16^ On these views, constraints on free movement and association directly influence the mind. But even one who rejects such views must surely accept that changes in one’s local environment can exert a direct causal effect on the mind, and that, in some cases, these effects might include conditions that impede agency—perhaps depression, hallucinations or paranoia. There is, for example, some evidence that solitary confinement and other forms of social or sensory deprivation can cause hallucinations and other perceptual disturbances.^17^

A final attempt to establish that bodily interference involves a more serious causal threat to agency than the restriction of free movement and association would appeal to the *means* via which these two interventions can impede agency. One might maintain that, even if constraints on free movement were to cause agency-impeding mental conditions such as depression or paranoia, there is still a sense in which they would be less threatening to agency than bodily interventions with similar affects. For it might be thought that, when environmental manipulations such as constraints on movement and association cause such conditions, they cause them only *through* engaging agential processes. The inmate subjected to solitary confinement only becomes depressed, or paranoid, as a result of *reflecting on* his situation. By contrast, when one interferes with the body of another, one may threaten his agency through purely brute causal mechanisms—for example, by biologically influencing the neural states on which the mind supervenes. Thus, bodily changes might perhaps be said to causally threaten agency both in their means of operation and their results, whereas environmental interventions threaten agency only in their results. And perhaps this difference justifies the claim that bodily interference constitutes a more serious causal threat to agency than do restrictions on free movement and association.

However, the distinction between agential and brute causal means does not map as neatly on to the distinction between bodily interference and constraints on movement and association as the above story suggests. Constraints on free movement and association can also affect the mind—and thus potentially threaten agency—through channels other than the engagement of agential processes. For example, it may be that the lack of diversity in physical and natural stimuli caused by incarceration has, through subconscious, nonagential mechanisms, an affect on brain structure, and thus on mental processing.^18^ Such effects would plausibly be as threatening to agency as the biological effects of drugs.

## Conclusions

The Robustness Claim maintains that the right to bodily integrity which protects against injections is more robust than the rights to free movement and association that protect against constraints on free movement and association of the sort involved in minimal incarceration. I have considered the possibility that we might defend this claim by appealing to case-based intuitions. But this strategy seemed unpromising. I noted that intuitions regarding the use of medical interventions *within the context of criminal justice* have little dialectic force, and may also have little evidential value, and once we moved away from the context of criminal justice, I was unable to identify any clear intuitive support for the Robustness Claim. I also considered the possibility that one might defend the Robustness Claim by showing that safe injections would (typically) (1) harm the victim of the interference more seriously than incarceration, or (2) constitute a more serious communicative or causal threat to agency. But I was unable to establish either result.

I do not claim to have refuted the Robustness Claim. There are no doubt many possible means of defending it that I have not considered, and that I am therefore not in a position to reject. Still, I believe that I have assessed the attempts to defend the robustness claim that are, at first sight, most promising. I thus hope that my arguments are sufficient to cast serious doubt on the Robustness Claim, and thus on the attempt to defend the Consent Requirement by appealing to a right to bodily integrity. These are doubts that have not yet been acknowledged in the debate about correctional medical interventions, where the Consent Requirement has simply been taken for granted.

How might a defender of the Consent Requirement respond to the challenge I have raised? One way to respond would be to seek to find an alternative way of showing that rights to bodily integrity are more robust than rights to free movement and association. Another possibility would be to seek to defend a finer grained right—for example, a right not to have one’s body interfered with *for certain reasons*. Perhaps, even if we have no highly robust right against bodily interference, we have a highly robust right against certain kinds of bodily interference—say, those that are motivated in certain ways. Yet another possible response—and the response that I believe to be most promising—would be to appeal not to a right to bodily integrity, but to a right to *mental* integrity, that is, a right against mental interference. In Sect. 6.2 above I argued that medical correctives need not, in virtue of the kind of bodily interference that they involve—namely, the injection of a drug—constitute a more serious causal threat to agency than minimal incarceration. I noted that injections need not, and often do not, have agency-undermining mental effects. However, medical correctives are not simply generic instances of the broader category of injections. One of the distinctive features of medical correctives—and something that sets them apart, for example, from medical forms of pandemic control—is that they are *intended* to have mental effects. They are intended to facilitate the offender’s rehabilitation. Their nonconsensual imposition might thus be thought to constitute a kind of nonconsensual *mental* interference. Moreover, it seems possible that we enjoy highly robust rights against such mental interference. After all, intentionally and nonconsensually altering someone’s mind might plausibly be thought to constitute a serious threat to agency.

These are thoughts that I cannot pursue in detail here. But I wish to suggest that it is not at all clear, in advance of detailed analysis, that an appeal to a right to mental integrity will succeed in buttressing the Consent Requirement. In contrast to the right to bodily integrity, the putative right to mental integrity enjoys no significant philosophical pedigree. Very little work has been done to determine whether there is such a right and, if so, what varieties of mental influence fall within its scope.^19^ (It seems very doubtful that there is any very general right against mental influence, for there are all sorts of ways in which we routinely influence the minds of others without, intuitively, violating their rights.) Much philosophical work would need to be done before we would be in a position to determine whether people generally enjoy a right against the kind of mental interference involved in the imposition of medical correctives, let alone a right sufficiently robust to retain its protective force following criminal offending.

Until this work has been done, it seems to me highly uncertain whether the Consent Requirement can be defended by reference to a right to mental integrity. Given this, and the problems I have raised for the attempt to defend the Consent Requirement by appealing to the right to *bodily* integrity, I believe that we should at least take seriously the possibility that the Consent Requirement is incorrect; that medical correctives could, at least in some cases, permissibly be provided without valid consent.

This would have at least two important implications. First, it would provide us with a second means of undermining the dominant objection to the use of medical correctives as an optional alternative to (further) incarceration. As we saw earlier, that objection maintains that offering a medical corrective in such circumstances is coercive, and this makes it impossible for the offender to validly consent to the corrective. The main response to this objection has been to maintain that, in fact, valid consent *can* be obtained in such circumstances. But I have suggested that, even if this response fails, there is a second line of defence; one could deny that valid consent is required in order to provide a medical corrective.

 Second, a failure of the Consent Requirement could have significant implications for the question whether the state may sometimes permissibly impose medical correctives compulsorily—that is, without giving the offender the option of refusing the intervention and remaining in prison. If the Consent Requirement is incorrect, then compulsory uses of medical correctives could *in principle* be justified. They would not be ruled out merely in virtue of their nonconsensual nature. However, it may not follow that the compulsory imposition of medical correctives is in fact justified. For there might be other moral reasons to prefer an approach in which medical correctives are offered as an optional alternative to (further) incarceration. Or it may be that, although consent is not *generally* required for medical correctives, it is required in some contexts—for example, where it can be obtained at little cost. Thus, though the argument presented in this paper raises the possibility that the compulsory imposition of medical correctives might be justified, it does not of itself establish as much.

## Footnotes

1 I take rehabilitation to consist in the improvement of the offender’s character, where ‘improvement’ can be understood narrowly, so as to imply only that the post-rehabilitation offender will be less likely to offend, or more broadly, so as to imply that the post-rehabilitation offender will be a morally better person.

2 For example, in the United Kingdom, methadone treatments have been imposed as part of Drug Treatment and Testing Orders imposed by the courts. See, for discussion, Eley et al. (2002) and Hough et al. (2003).

3 Other pharmaceuticals have also been used for this purpose. For example, selective serotonin re-uptake inhibitors, a widely used class of anti-depressants, have been used to prevent recidivism as they have, as a common side-effect, the reduction of libido. Historically, physical destruction of the testes (known as physical or surgical castration) was also widely used to prevent sexual recidivism. See, for a discussion of medical interventions used to prevent sexual recidivism, Thibaut et al. (2010).

4 I will not distinguish between cases in which the medical corrective, though used primarily to achieve a rehabilitative goal, also achieves (or is intended to achieve) some orthodox therapeutic purpose such as the treatment of a disease, and cases where it does not. I believe that the arguments that I will offer apply equally to both cases. Additionally, I will not have anything to say about the use of medical interventions to achieve other forensic goals such as deterrence, retribution or incapacitation. I believe that the arguments that I discuss in this paper would bear also on such practices, but I allow that these practices may be susceptible to objections that I do not consider here.

5 For a classic statement of the view that rehabilitation is the sole goal of incarceration, see de Beaumont and de Tocqueville (1833, 17–18).

6 Perhaps the most commonly heard defence of a consent requirement within clinical medicine appeals not to a right to bodily integrity, but to a right to autonomy. But we clearly do not enjoy a right to autonomy over all aspects of our life. For example, I do not enjoy a right to autonomously control the behaviour of others around me. I take it that when one appeals to a right to bodily autonomy to defend a consent requirement in medicine, one is appealing to a right to autonomously control what forms of bodily interference one is subject to. And this, I take it, is parasitic on a more basic right to bodily integrity which rules out (most cases of) nonconsensual bodily interference.

7 Similar points could also be made regarding external property rights and rights to control of one’s own time and labour. It might seem that, if criminal offending can make one liable to fines, probation regimens or community service, it must cause these rights to lose some of their normal protective force, for these rights would rule out the imposition of such interventions on non-offenders.

8 I assume that rights to free movement and free association are the only rights that protect against the imposition of minimal incarceration.

9 I assume, for simplicity, that loss of protective force is a binary matter. A more sophisticated account of the robustness of rights could allow that the protective force offered by a right varies as a matter of degree.

10 I assume that this right provides equal protection against all forms of bodily interference that fit this description.

11 It is not clear precisely how much distress such constraints could be expected to cause in the absence of negative effects on life projects or relationships. It is reported that existing incarceration practices frequently cause significant distress in inmates. See, for example, Haney (2003). However, this distress could be due to the actual damage to relationships and life projects caused by incarceration, or to features of prevailing incarceration conditions that are distinct from the restrictions on movement and association that it entails— for example, it might be due to the perceived risk of being mistreated by guards or other inmates.

12 See, for development of this point and its significance for the morality of bodily interference, Eyal 2009.

13 A less subjective version of this argument would maintain that interference with the body is itself harmful even leaving aside the psychological distress it causes. The psychological pain that such interferences cause is not the primary harm, but is rather due to our perceiving a more objective harm. I do not separately consider this argument, but I believe that the arguments that I offer below in respect of its more subjective variant apply equally to it.

14 It is possible, of course, that young people and healthcare workers are not representative of the wider population in the way that they experience compulsory or costly-to-avoid safe injections. It may be that people generally experience the imposition of safe injections very negatively, though young people and healthcare workers do not. It is also possible that the lack of research on and public debate about the way in which such interventions are experienced reflects a failure to notice or appropriately respond to the way in which they are experienced, rather than a correct assumption that they are not experienced in seriously harmful ways. For these reasons, the absence of research and debate on this matter constitutes only weak evidence fro the view that compulsory (or costly to avoid) injections do not typically cause substantial experiential harms.

15 To say that that an intervention is a causal threat to agency is, perhaps, to say that it risks inflicting a certain kind of *harm* (the harm of having one’s agency undermined). Nevertheless, an attempt to explain the robustness of a right by maintaining that it protects against serious causal threats to agency is interestingly distinct from the attempt to explain it by maintaining that the right protects against seriously harmful forms of interference. If it is ultimately the (typical) harmfulness of an intervention that determines the robustness of the right we enjoy against it, then we would enjoy a more robust right against an intervention that is all-things-considered more harmful, though a less serious casual threat to agency, than another. If it is the seriousness of the causal threat to agency that matters, the situation will be reversed.

16 See, for the classic statement of the extended mind thesis, Clark and Chalmers (1998). For a discussion of some of it’s implications for the ethics of brain-influencing biomedical interventions, see Levy (2007).

17 See for a review, Haney (2003). Note, however, that these findings have been contested. For some contrasting results, see O’Keefe et al. (2010).

18 It is not clear, for example, that the sensory disturbances and hallucinations which have been attributed to solitary confinement can plausibly be attributed to agential mechanisms. There is also evidence that social isolation can have significant neurological effects in non-human mammals, in which the effects could not plausibly be attributed to agential mechanisms. This is suggestive that human brains might also by influenced by social isolation through nonagential mechanisms. See, for example, Heidbreder et al. (2000); Weiss et al. (2004); and Wongwitdecha and Marsden (1996).

19 For an initial exploration of the right to mental integrity, see Bublitz & Merkel (2014).
